# Editorial: Hair Follicle Stem Cell Regeneration in Aging

**DOI:** 10.3389/fcell.2021.799268

**Published:** 2021-11-25

**Authors:** Mingxing Lei, Sung-Jan Lin, Cheng-Ming Chuong

**Affiliations:** ^1^ 111 Project Laboratory of Biomechanics and Tissue Repair, College of Bioengineering, Chongqing University, Chongqing, China; ^2^ Key Laboratory of Biorheological Science and Technology of the Ministry of Education, College of Bioengineering, Chongqing University, Chongqing, China; ^3^ Department of Biomedical Engineering and Department of Dermatology, College of Engineering and College of Medicine, National Taiwan University, Taipei, Taiwan; ^4^ Department of Dermatology, National Taiwan University Hospital, Taipei, Taiwan; ^5^ Department of Pathology, Keck School of Medicine, University of Southern California, Los Angeles, CA, United States

**Keywords:** aging, hair regeneration, niche, skin organoid, wound-induced hair neogenesis

## Hair Follicle Stem Cell Regeneration in Aging

**FIGURE 1:**
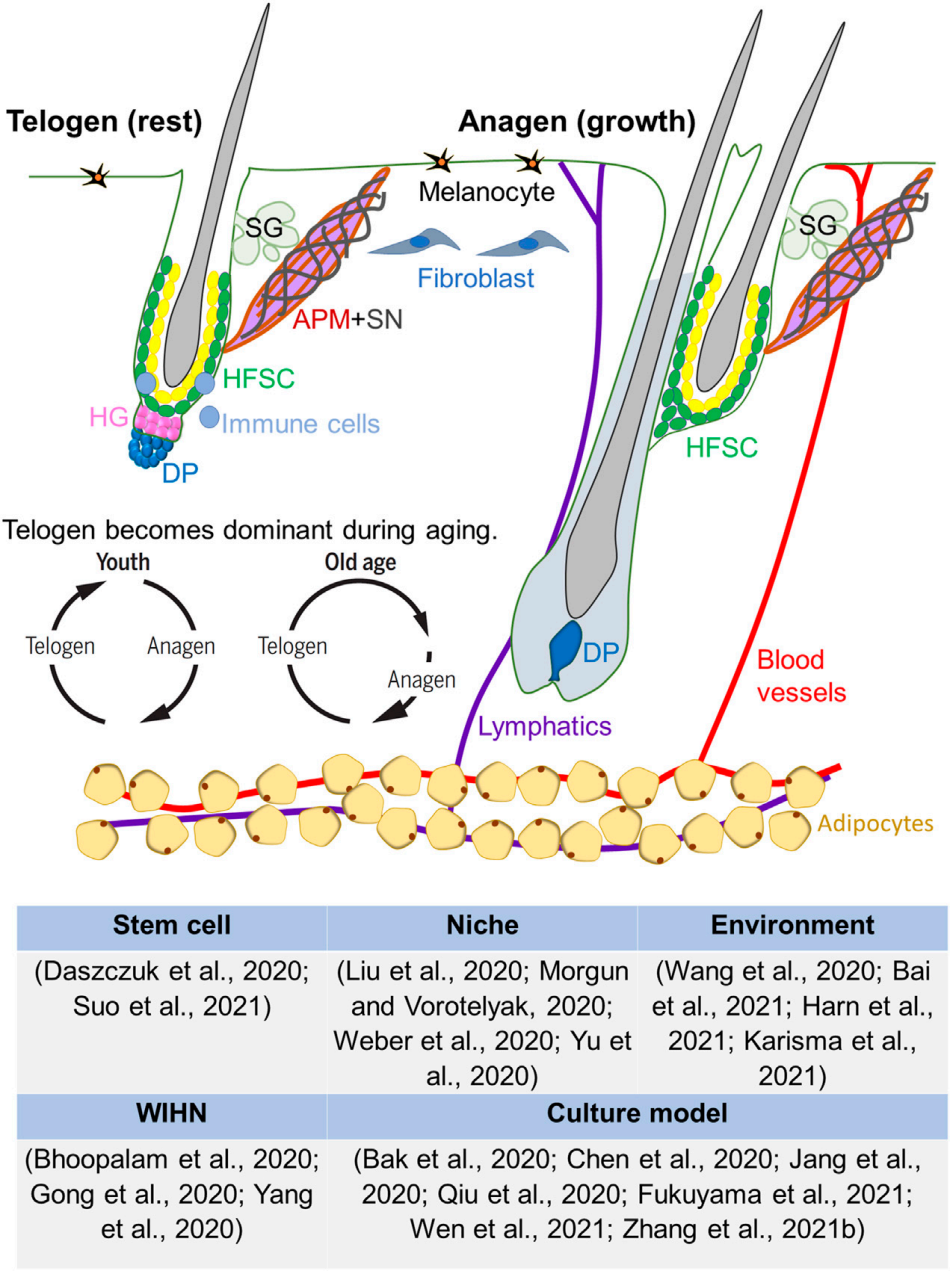
Hair follicle stem cell regeneration in aging. Hair follicle undergoes cyclic renewal, which is regulated by cyclic activation of quiescent stem cells controlled by a core circuit involving beta-catenin. This core molecular circuit involves interactions between hair follicle stem cell (HFSC) and dermal papilla within the hair follicle. Adjacent to the follicles, blood vessels, nerves, arrector pili muscle, intradermal adipose tissues, etc. form a larger niche that also can affect HFSC activation. Further, outside environment can also modulate the cyclic renewal behavior of hair follicles. The effects of these modulators change during aging. The telogen phase of the hair cycle becomes dominant during aging, leading to decreased hair regeneration. Aged cells can be reactivated to regenerate hair follicles under wound-induced hair neogenesis condition or using several 2D or 3D culture models. APM, arrector pili muscle; DP, dermal papilla; HFSC, hair follicle stem cells; HG, hair germ; SG, sebaceous gland; SN, sympathetic nerve.

With stem cells that can be activated and silenced cyclically, hair follicle experiences multiple rounds of growth phase (anagen), regression phase (catagen), and resting phase (telogen) during lifespan. Hair cycling is initiated by cyclic renewal or physiological cyclic regeneration of stem cells. However, tissues and organs undergo structural and functional declines in the aging process, with physiological and pathological changes regulated by intrinsic and extrinsic factors that dictate the cell fate (Lei and Chuong, 2016) (Figure 1). As one of the important appendages of the skin, the hair follicle is a complex mini-organ with visible signs, such as decreased regenerative ability that leads to alopecia, and hair graying due to less melanin production by melanocyte stem cells during aging (Qiu et al., 2019). Hair regenerative ability is gradually decreased because hair follicle stem cells enter a long quiescent state (Chen et al., 2014), or differentiate into other skin epithelial lineages (Matsumura et al., 2016), or escape from the hair follicle niche during aging (Zhang C. et al., 2021). These features promote hair follicle to become a widely used model for studying regeneration. As over 30% of the population all over the world suffer from partial or complete hair loss, particularly most people undergo alopecia during aging, understanding the mechanism by which hair follicle changes during aging is of great interest in regenerative biology and is essential for regenerative medicine.

The field of hair follicle biology has expanded tremendously with broader scales and diverse approaches that aim to elucidate the mechanisms of hair follicle regeneration, as well as to devise potential therapeutic strategies to delay the aging processes or restore the regenerative ability. Here we organize this Research Topic with a collection of original research and review articles that explore aging-related hair follicle stem cell regeneration. Although not fully covering the comprehensive hair follicle stem cell field, this issue provides new insight into hair regeneration from different perspectives, mainly at the physiological, wound healing, and *in vitro* cultivation levels.

Skin which covers the surface of our human body is constantly subjected to environmental insults. Ultraviolet (UV) radiation is the major cause of skin aging that is called photoaging, which provokes oxidative stress, inflammatory responses, and DNA damage. Three papers in this Research Topic focus on studying the role of UVA in skin aging (Wang et al., 2020; Bai et al., 2021; Karisma et al., 2021). Bai et al. investigated rapamycin as a macrolide immunosuppressant that protects skin fibroblasts from UVA-induced photoaging (Bai et al., 2021). They found that rapamycin-treated human dermal fibroblasts have a decreased expression of p53 and phosphorylated HSP27, as well as reduced genotoxic and oxidative stress levels. Wang et al. identified purified vitexin compound 1 as a novel lignanoid that attenuates UVA-induced senescence in human dermal fibroblasts *in vitro* and mouse skin *in vivo*, through repressing UVA-induced phosphorylation of MAPK1 (Wang et al., 2020). Although UVA has a harmful effect to cause skin photoaging, it is a double-edged sword that can be used as a stimulus to switch the photo-controlled drug release upon a “switch on-switch off” procedure, which can be developed for clinical use. A review comprehensively summarized how UVA-triggered drug release as a promising potential for skin photoprotection and phototherapy (Karisma et al., 2021). In addition to UVA-induced photoaging, recent advancements suggest that tissue mechanics is largely involved in regulating skin aging. Another review by Harn et al. described the manifestations of the aging skin, and fully summarized how tissue mechanics influences extracellular matrix dysregulation, wrinkling, and wound healing during skin aging (Harn et al., 2021). They also proposed how perturbed tensional homeostasis impacts physiological functioning of the skin, including how skin tension influences hair regeneration and wound healing. Then, they enumerated several potential chemical and mechanical approaches that may be used for developing new drugs against skin aging.

Hair follicle is one of the skin appendages that is mostly influenced by aging. Hairs are gradually lost during the aging process, which is largely controlled by intrinsic epigenetic/genetic status and extrinsic environmental stimuli that regulate the hair follicle stem cell behavior during hair cycling. Decreased expression of activators and increased expression of inhibitors lead to decreased or failed hair regeneration in aged skin, which may cause alopecia eventually. The telogen phase of the hair follicle, particularly the refractory telogen phase becomes longer and longer, compared to the competent telogen phase during aging. In this Research Topic, Suo et al. identified that different levels of Vitamin A have different roles in regulating hair cycling. High-level of dietary retinyl esters results in a greater percentage of hair follicles in refractory telogen, and vice versa (Suo et al., 2021). They showed that Vitamin A regulates hair follicle stem cell activity through BMP and Wnt signaling pathways. This study identified a new intrinsic factor that regulates hair follicle stem cell activity. Two reviews summarized the recent progress on how intrinsic and extrinsic factors modulate hair follicle stem cells activities. Daszczuk et al. discussed the current views on the intrinsic molecular mechanisms that regulate cyclic hair regeneration (Daszczuk et al., 2020). They proposed an intrinsic oscillation of gene networks model in control of hair cycling, which has potential instruction for translational regenerative medicine. Morgun and Vorotelyak reviewed how inflammation as one of the extrinsic factors that regulates hair cycling, re-epithelialization, and wound-induced hair neogenesis (Morgun and Vorotelyak, 2020). They also discussed how inflammation influences hair follicle stem cell activation during wound healing at the cellular and molecular levels. These two reviews developed further the concept that synergistic action of intrinsic and extrinsic factors is essential for proper hair regeneration.

Dermal papilla (DP) is the signaling center for regulating hair growth and regeneration. In this Research Topic, comparative transcriptome analyses by Weber et al. identified a Wnt agonist, R-spondin-1, is significantly decreased in the DP of adult human hair follicles compared to that of the fetal scalp hair follicles (Weber et al., 2020). Adult cells which lose the regenerative ability can be partially restored to regenerate hair follicles with addition of R-spondin-1 recombinant protein. Another research by Yu et al. showed that Twist1 functions on regulating hair follicle induction ability in the DP by forming a ternary complex with Tcf4 and b-catenin, which delays the aging process of the DP cells by Tcf4-mediated Wnt/b-catenin signaling pathway (Yu et al., 2020). Under pathological conditions such as androgenetic alopecia (AGA) which is occurring in more than half of men aged over 50 years old, hair follicles are attacked by dihydrotestosterone and then become miniaturized. Liu et al. identified that Zyxin, an actin-interacting protein, is increased in the DP of AGA-affected frontal hair follicles compared to that of the unaffected occipital hair follicles (Liu et al., 2020). Knockout of Zyxin leads to enhanced hair growth and anagen reentry, indicating that Zyxin is an inhibitor for hair regeneration. This offers a potential therapeutic target for treating AGA.

As skin is located at the outmost of our body, it is vulnerable to be injured by mechanical, chemical, or biological insults. Research conducted by Yang et al. used a promising new method called photodynamic therapy to treat mouse skin wounds infected with *Pseudomonas aeruginosa* (Yang et al., 2020). They showed that 5-aminolevulinic acid photodynamic therapy is beneficial for eliminating *Pseudomonas aeruginosa* and accelerating skin wound healing in mice, by modulating the expression of inflammatory factors (TNF-a and IL-1b) and growth factors (TGF-b1 and VEGF). Since the establishment of the wound-induced hair neogenesis model by Ito et al., in 2007 (Chuong, 2007; Ito et al., 2007), a number of following studies aimed to study the molecular mechanisms by which *de novo* hairs regenerate at the center of the wound bed during healing of large cutaneous wounds. Bhoopalam in this Research Topic reviewed the recent progress in digging the underpinnings of wound-induced hair neogenesis, at the cellular (hair follicle stem cells, fibroblasts, inflammatory cells) and molecular (Wnt/b-catenin, Hedgehog, dsRNA/IL-6/STAT3/Retinoic Acid, Hippo signaling pathways) levels (Bhoopalam et al., 2020). They also proposed several wonderful interesting questions that remain to be further investigated in this field. Besides, this issue also included one research paper which identified IL-36a as a new factor modulating the outcome of wound-induced hair neogenesis (Gong et al., 2020). They show that IL-36a is increased during wound healing, and application of recombinant murine IL-36a protein into large skin wound promotes wound-induced hair neogenesis, probably through regulating the IL-6/STAT3 pathway.

Scientists also have been trying to restore hair regeneration using *in vitro* culture models, with focuses on modulating hair follicle stem cells and DP cells. Bak et al. in this Research Topic transplanted the cultured human outer root sheath (ORS) cells along with freshly isolated neonatal mouse dermal cells to the nude mice, and observed that the long-term (42 days) cultured ORS cells have a decreased trichogenicity compared to the short-term (20 days) cultured ORS cells (Bak et al., 2020). They identified that FOXA2 in ORS cells functions in regulating the trichogenicity of human ORS cells. Wen et al. revealed that inhibition of Rho-Associated Protein Kinase by a specific inhibitor Y-27632 can increase the adhesion, proliferation, and stemness of the primary cultured human hair follicle stem cells through the ERK/MAPK pathway (Wen et al., 2021). As two-dimensional (2D) cultured cells quickly lose their regenerative ability, most researches turned to modulate hair regeneration under 3D culture conditions. Zhang et al. compared the gene expression in 2D and 3D cultured DP cells treated with dihydrotestosterone, which can inhibit proliferation but induce apoptosis in DP cells co-cultured with ORS cells (Zhang Y. et al., 2021). Without dihydrotestosterone treatment, the ORS cells showed significant proliferation when co-cultured with DP cells. They used RNA-sequencing to identify that extracellular matrix synthesis is increased in 3D culture DP cells, and set up a transcription factor–miRNA coregulatory network in this co-culture system, which may be beneficial for future AGA studies at the molecular level. In addition to the extracellular matrix, the same group also identified that miR-140-5p in small extracellular vesicles secreted by DP cells suppresses Bmp2 expression to promote proliferation of ORS and hair matrix cells in a co-culture model (Chen et al., 2020). Tissue engineering models have been established to promote hair follicle neogenesis. One research article by Fukuyama et al. set up a reconstitution assay, in which human-induced pluripotent stem cell-derived DP cell aggregates were co-cultured with human keratinocytes in a 3D cylindrical structure inserted with a fragment of guide nylon fiber (a mimic hair fiber). They observed morphologically resembled hair follicle structure that expresses hair follicle-related genes in the 2-week culture (Fukuyama et al., 2021). This study adds a new tissue engineering model for hair regeneration, based on the principle of epithelial-mesenchymal interaction.

*In vitro* culture models have been established for drug screening. Jang et al. set up a 3D co-culture system, in which human DP cells and ORS cells were cultured in an ultra-low attachment plate, and formed a “two-cell assemblage” structure that can be used as a model to test the hair growth-promoting effect of library molecules (Jang et al., 2020). Epithelial-mesenchymal interaction also leads to the formation of a skin cyst when mouse epidermal and dermal cells were mixed and 3-dimensionally cultured on a transwell insert, namely organoid culture (Lei et al., 2017). The dissociated single cells can self-organize and undergo a series of morphological transitions, forming a planar hair-bearing skin through aggregation, polarization, coalescence, and planarization stages. Compared to the clinically diagnosed skin cyst, we proposed that the skin cyst forming during skin organoid culture is not a pathological dead-end, but harbors the potential to regenerate skin appendages such as hair follicle and sebaceous gland (Qiu et al., 2020).

In conclusion, recent understanding of aging includes stem cell exhaustion, cellular senescence, altered intercellular communication, epigenetic alterations, genomic instability, telomere attrition, loss of proteostasis, deregulated nutrient sensing, mitochondrial dysfunction, environmental stresses, etc. These changes lead to obvious features in the skin such as hair loss and graying, skin wrinkling, age spots, loss of elasticity, laxity, sagging, and rough-textured appearance (Harn et al., 2021). The experimental discoveries and perspectives presented in this Research Topic add new layers for understanding hair follicle stem cell regeneration during aging. With cutting-edge experimental methodologies and ever-increasing cross-discipline approaches employed in this Research Topic, we witness the robust progress in this field. We hope this Research Topic will pave new ways for elucidating new mechanisms of aging-related hair follicle stem cell regeneration, and also inspire the development of new therapeutic strategies for treating aging-related hair disorders with this momentum.
